# Peer Referral Pathways and 6-Month Outcomes in a Tirzepatide-Supported Digital Weight Loss Service: Retrospective Cohort Study

**DOI:** 10.2196/105583

**Published:** 2026-09-28

**Authors:** Louis Talay, Laura Swinckels, Jason Hom, Marilyn Tan, Neera Ahuja

**Affiliations:** 1 Department of Clinical Research, Eucalyptus Health, Level 3, 155 Clarence Street, Sydney, New South Wales, 2006, Australia, 61 424 696379; 2 Department of Medicine, Stanford University Medical Centre, Stanford, CA, United States

**Keywords:** real-world obesity program, peer-to-peer network, tirzepatide, telehealth, digital weight loss service

## Abstract

In a retrospective analysis of 34,449 adults using an unsubsidized, tirzepatide-supported digital weight loss service (DWLS) in Australia, patients entering via peer referral showed higher 6-month program adherence and greater percentage weight loss than propensity score–matched nonreferred patients, suggesting that peer referral pathways may support retention and effectiveness in medicated obesity care.

## Introduction

The real-world effectiveness of modern weight loss medications, such as tirzepatide and semaglutide, depends heavily on medication adherence and sustained behavioral modification [1]. Recent studies have demonstrated that 6- and 12-month adherence rates in real-world tirzepatide-supported obesity programs are generally low [2,3]. While digital weight-loss services (DWLSs) deploy algorithmic nudges and multidisciplinary coaching to drive retention, external sociopsychological factors, such as preexisting peer support networks, are frequently overlooked [4]. Behavioral medicine frameworks suggest that patients entering interventions via social referrals benefit from built-in accountability and shared social proof, which can mitigate the attrition commonly observed in remote care [5,6]. However, the specific impact of peer-to-peer referral pathways on adherence and objective weight loss metrics within a medication-supported DWLS remains unquantified. This study evaluated the programmatic persistence and 6-month percentage weight loss of referred vs nonreferred patients receiving tirzepatide treatment via an Australian DWLS.

## Methods

### Background

This retrospective cohort study examined deidentified data from the Juniper (Eucalyptus Health) DWLS data repository on Google BigQuery. Patients were included if they initiated a tirzepatide treatment pathway between May 20 and December 2, 2025. Juniper patient eligibility is determined by doctors and nurse practitioners in accordance with Therapeutic Goods Administration guidelines for obesity treatment. Once accepted, patients communicate with prescribing clinicians, health coaches, and medical support staff via the Juniper app. All patient data are stored in the Juniper data repository, including weight data, which are submitted by patients via Bluetooth smart scales or manual input on the Juniper app.

The study cohort was stratified into referred patients and nonreferred patients. Referral was implemented as a tracked marketing mechanism using invitation links that existing patients could share with others. When a new patient enrolled via this link, the referrer and referee received a discount of AUD $75 (AUD $1=US $0.65 as of December 1, 2025) on 2 of their upcoming tirzepatide orders. The referral program did not include an in-app chat function or a peer-support group. Order costs ranged from AUD $449 to $855 (depending on tirzepatide dose) over the study period.

### Adherence Criteria

Program adherence at 6 months was operationalized using 2 measures:

- Medication adherence: fulfillment of 5 or more orders within 183 days of program initiation.
- Per-protocol persistence: submission of at least 1 verified body weight entry within a strict window of day 173 to day 193 postinitiation.

### Covariates

To isolate the independent effect of peer referral on mean 6-month percentage weight loss, propensity score matching (PSM) was implemented. Referred patients with a minimum 6-month program tenure were matched 1:1 with nonreferred controls using a nearest-neighbor algorithm based on age, sex at birth, ethnicity, baseline BMI, comorbidity count, previous tirzepatide/semaglutide use, and first order price. Differences in adherence rates were evaluated using Pearson *χ*^2^ tests, and matched weight loss outcomes were compared via independent *t* tests. For differences in ordinal variables such as comorbidity count, we used a Wilcoxon rank sum test. To account for multiple statistical testing across baseline characteristics and outcome metrics, *P* values were adjusted using the Benjamini-Hochberg false discovery rate procedure. Statistical analyses were performed in RStudio (version 2023.06.1; Posit PBC), with significance set at *P*=.05.

### Ethical Considerations

The Stanford University Institutional Review Board determined that this study did not constitute human subjects research (protocol 82970, October 22, 2025).

## Results

The full cohort comprised 2576 referred and 31,873 nonreferred patients. Age, BMI, and ethnicity distributions were similar between groups (Table 1). However, the referred cohort organically exhibited a significantly higher concentration of male patients (592/2576, 23%) than the nonreferred cohort (2486/31,873, 7.8%) (*P*<.001) and a lower prevalence of prior tirzepatide/semaglutide use (438/3576, 17%) than the nonreferred cohort (7331/31,873, 23%) (*P*<.001).

**Table 1.** Baseline characteristics of adults enrolled in an Australian tirzepatide-supported digital weight loss service by referral status (referred vs nonreferred), May-December 2025.

Variable	Referred cohort (N=2576)	Nonreferred cohort (N=31,873)	Adjusted *P* (FDR)^a^
Sex at birth, n (%)			<.001
Female	1984 (77)	29,387 (92.2)	
Male	592 (23)	2486 (7.8)	
Ethnicity binary, n (%)			.17
White	2128 (82.6)	26,040 (81.7)	
Non-White	448 (17.4)	5833 (18.3)	
Initial BMI, mean (SD)	33.5 (5.27)	33.3 (5.49)	.16
Age category, n (%)			.20
Under 30 years	314 (12.2)	4474 (14)	
30‐44 years	1249 (48.5)	14,907 (46.8)	
45‐59 years	848 (32.9)	11,072 (34.7)	
60+ years	165 (6.4)	1420 (4.5)	
Total comorbidities, n (%)			.05
0 complications	1571 (61)	18,486 (58)	
1 complication	799 (31)	10,200 (32)	
2 complications	154 (6)	2550 (8)	
3+ complications	52 (2)	637 (2)	
Prior tirzepatide/semaglutide use, n (%)			<.001
Yes, prior history	438 (17)	7331 (23.0)	
Subscription plan, n (%)			.14
Standard monthly	1958 (76)	25,179 (79)	
Bundled package	618 (24)	6694 (21)	
Month 1 weight track count, n (%)			.16
Baseline only	108 (4.2)	1787 (5.6)	
2‐5 entries	693 (26.9)	7885 (24.7)	
6‐10 entries	446 (17.3)	6271 (19.7)	
11‐15 entries	513 (19.9)	5688 (17.8)	
16‐25 entries	374 (14.5)	5302 (16.6)	
>25 entries	442 (17.2)	4940 (15.5)	
Financial markers (median)			.30
First-order price ($ AUD^b^)	385	390	

a FDR: false discovery rate.

b AUD $1=US $0.65 as of December 1, 2025.

A total of 9508 (27.6%) patients met the study’s 6-month adherence criteria (Table 2). Of the remaining 24,941 patients, 10,318 (30%) failed to receive at least 5 tirzepatide orders, while 14,623 (42.4%) met this criterion but failed to submit data within the specified window. Referred patients achieved significantly higher 6-month medication adherence (≥5 orders) than nonreferred patients (1236/2576 [48%] vs 13,387/31,873 [42%]; *P*<.01). This adherence disparity was maintained under the per-protocol persistence criteria, with 35% of referred patients sustaining medication compliance and submitting data within the 6-month weight window versus 27% of the nonreferred cohort (*P*<.01). Following 1:1 PSM to control for baseline imbalances (n=804 matched pairs), all mean SDs had values <0.10, indicating good covariate balance. Referred patients achieved a mean weight loss of 16.2% (SD 6.2%) compared to 14.1% (SD 6.5%) in the matched nonreferred control group (*P*<.001).

**Table 2.** Six-month medication adherence and clinical weight loss outcomes among adults in the Juniper Australia tirzepatide-supported digital weight loss service in the nonreferred (propensity score–matched) cohort, May-December 2025.

Evaluation metric	Referred cohort	Nonreferred cohort	Effect size (95% CI)	Adjusted *P* value (FDR^a^)
Per protocol adherence matrix	n*=*902	n*=*8606	OR^b^=0.99 (0.91‐1.07)	.78
6-month matched weight loss (PSM^c^)	n*=*804	n*=*804	—^d^	—
Mean percentage weight loss, % (SD)	16.2% (6.2%)	14.1% (6.5%)	Difference=2.10 (1.56‐2.64)	<.001
Achieved ≥5% total weight loss, n (%)	756 (94%)	724 (90%)	OR=1.74 (1.20‐2.52)	.01
Achieved ≥10% total weight loss, n (%)	675(84%)	535 (66.5%)	OR=2.63 (2.07‐3.34)	<.001
Achieved ≥15% total weight loss, n (%)	498 (62%)	386 (48%)	OR=1.76 (1.45‐2.15)	<.001

a FDR: false discovery rate.

b OR: odds ratio.

c PSM: propensity score matching.

d Not applicable.

## Discussion

In this retrospective analysis of a tirzepatide-supported DWLS, patients entering via peer referral were more likely to remain engaged in the program and, in PSM analyses, had higher observed 6-month percentage weight loss than nonreferred patients (16.2% vs 14.1%). These findings are preliminary but suggest that peer referral networks may have an important role in supporting retention and effectiveness in unsubsidized, medication-supported DWLSs.

Notably, the referral pathway was associated with a shift in participant demographics, particularly in the higher male representation (23% vs 7.8%). As men are traditionally underrepresented in weight management services [7], peer referral mechanisms may offer a promising approach to engage this group. However, further work is needed to understand underlying motivations and barriers.

This work has several limitations. Its retrospective, observational design limits causal inference, and although PSM was used to reduce demographic variability, residual confounding by unmeasured variables (eg, intrinsic motivation, socioeconomic status, or social network structure) is likely [8,9]. The analysis of weight outcomes in a per-protocol framework introduced survivorship bias and may overestimate effects relative to a broader DWLS population. Additionally, all weight data were self-reported and were therefore susceptible to error and social desirability bias. Finally, this study reflects a single commercial, unsubsidized Australian DWLS, which may limit generalizability to other health systems or populations. The observed associations cannot disentangle tirzepatide’s direct pharmacological effects from digital support components or social factors related to referral.

Despite these constraints, the data suggest that peer referral pathways may improve program persistence and weight loss within a medicated DWLS. Prospective studies are needed to clarify mechanisms, confirm reproducibility, and determine how best to operationalize peer networks to enhance retention and outcomes in obesity care.

## Abbreviations

DWLS: digital weight loss service
PSM: propensity score matching
